# Heavy Metal-Induced Retinal Injury in Zebrafish (*Danio rerio*): Histopathological Alterations, Molecular Mechanisms and Regenerative Responses

**DOI:** 10.3390/toxics14090772

**Published:** 2026-08-30

**Authors:** Alina Iliuța Olărița, Alexandra Szilagyi, Alexandra Jităreanu, Gheorghe Solcan, Carmen Solcan

**Affiliations:** 1 Faculty of Veterinary Medicine, “Ion Ionescu de la Brad” Iasi University of Life Sciences, 8 M. Sadoveanu Alley, 700489 Iasi, Romania; olarita.alinailiuta@yahoo.com (A.I.O.); alexandra.szilagyi13@yahoo.com (A.S.); carmen.solcan@iuls.ro (C.S.); 2 Faculty of Pharmacy, University of Medicine and Pharmacy “Grigore T. Popa” Iasi, 700115 Iasi, Romania

**Keywords:** zebrafish, *Danio rerio*, heavy metals, retinal toxicity, ocular toxicology, retinal degeneration, oxidative stress, photoreceptors, Müller glia, retinal regeneration

## Abstract

Heavy metal contamination is a significant environmental risk factor for ocular dysfunction and retinal degeneration in both aquatic organisms and humans. Zebrafish (*Danio rerio*) have become an important vertebrate model for retinal toxicology research due to their conserved retinal architecture, cone-rich visual system, optical transparency during development, and remarkable capacity for retinal regeneration. This narrative review synthesizes current evidence on the effects of heavy metals on the development, structure, function, and regenerative responses of the zebrafish visual system, with particular focus on retinal alterations, molecular mechanisms, and visual impairment. Studies indicate that exposure to cadmium, lead, arsenic, chromium, copper, mercury, and metal mixtures results in retinal disorganization, photoreceptor degeneration, vacuolization, retinal pigment epithelium damage, impaired retinogenesis, and alterations in visually mediated behaviors. Mechanistic investigations reveal that heavy metal-induced retinal toxicity involves oxidative stress, mitochondrial dysfunction, endoplasmic reticulum stress, apoptosis, neuroinflammation, DNA damage, and dysregulation of genes critical for retinal development and photoreceptor maintenance. Functional assays, such as the optomotor response and optokinetic reflex, provide sensitive endpoints for detecting visual dysfunction that may precede overt structural degeneration. Furthermore, the unique regenerative capacity of the zebrafish retina, primarily mediated by Müller glia reprogramming, provides valuable opportunities to study endogenous retinal repair following toxic injury. Overall, current evidence establishes zebrafish as a versatile and translationally relevant model for investigating heavy metal-induced retinal injury, visual dysfunction, and regenerative responses. Future research that incorporates environmentally relevant exposure paradigms, mixture toxicology, multi-omics approaches, and regenerative signaling pathways is likely to enhance understanding of metal-associated retinal disease and support the development of novel therapeutic strategies for retinal degeneration and vision loss.

## 1. Introduction

Environmental pollution represents a critical global challenge, threatening both ecosystem integrity and human health. Heavy metals are particularly concerning among environmental contaminants due to their persistence, bioaccumulation, widespread distribution, and ability to cause long-term biological effects even at relatively low concentrations. Anthropogenic activities such as mining, metal processing, industrial manufacturing, agriculture, fossil fuel combustion, and improper waste disposal have significantly increased environmental concentrations of toxic metals, including cadmium (Cd), lead (Pb), arsenic (As), chromium (Cr), mercury (Hg), and copper (Cu) [1,2,3]. Consequently, aquatic organisms are continuously exposed to complex mixtures of metals which accumulate in tissues and disrupt various physiological and cellular processes [1].

The visual system is highly susceptible to toxic injury due to its high metabolic activity, elevated oxygen consumption, continuous exposure to light, abundance of polyunsaturated fatty acids, and relatively limited antioxidant defenses [4,5]. The retina, a highly specialized neural tissue responsible for phototransduction and visual signal processing, is particularly vulnerable to oxidative stress, mitochondrial dysfunction, inflammation, and apoptotic cell death induced by environmental toxicants [6,7,8]. In humans, chronic exposure to heavy metals has been linked to a wide range of ocular disorders, such as retinal degeneration, optic neuropathy, age-related macular degeneration, visual impairment, and neurodevelopmental abnormalities affecting the visual system [7,8]. Although these adverse effects are increasingly recognized, the molecular and cellular mechanisms underlying heavy metal-induced retinal toxicity are not yet fully understood.

Over the past two decades, the zebrafish (*Danio rerio*) has become a widely recognized vertebrate model for research in developmental biology, toxicology, neuroscience, and ophthalmology. Key experimental advantages include external fertilization, rapid embryonic development, high fecundity, optical transparency during early developmental stages, and significant genetic homology with humans [9,10,11]. In addition to heavy metal research, zebrafish have been widely utilized to examine the neurotoxic effects of various environmental contaminants, such as pesticides, pharmaceuticals, and micro- and nanoplastics. This demonstrates the model’s versatility for investigating conserved mechanisms of environmentally induced neurotoxicity and sensory dysfunction [12]. Importantly, the zebrafish eye exhibits the principal anatomical and functional features of the vertebrate visual system, including a multilayered retina, retinal pigment epithelium, photoreceptors, and conserved visual processing pathways [10,11,13]. Moreover, unlike traditional rodent models, zebrafish are diurnal and possess a cone-rich retina that more closely resembles the human photopic visual system, making them particularly valuable for studies of retinal injury and visual dysfunction [14,15,16,17].

The zebrafish retina is characterized by a remarkable regenerative capacity. After retinal injury, Müller glial cells dedifferentiate, proliferate, and generate retinal progenitor cells that can replace damaged neurons, including photoreceptors and retinal ganglion cells [18,19,20,21,22]. This unique regenerative response provides an exceptional opportunity to investigate both the mechanisms underlying toxicant-induced retinal degeneration and the endogenous cellular and molecular pathways involved in retinal repair and functional recovery.

A growing body of experimental evidence indicates that heavy metal exposure induces a wide range of structural, molecular, and functional alterations in the zebrafish visual system. Documented effects include microphthalmia, retinal disorganization, photoreceptor degeneration, retinal pigment epithelium damage, vacuolization, impaired retinogenesis, oxidative stress, apoptosis, neuroinflammation, and deficits in visually mediated behaviors [23,24,25,26,27,28,29]. Collectively, these findings establish zebrafish as a robust and translationally relevant model for elucidating the mechanisms of environmental ocular toxicity and their implications for human retinal disease.

This narrative review provides a comprehensive overview of current knowledge on heavy metal-induced retinal toxicity in zebrafish. It emphasizes ocular anatomy, histopathological alterations, molecular mechanisms of retinal injury, visually mediated behavioral deficits, retinal regeneration, and emerging histopathological and multi-omics approaches for investigating ocular toxicity. By integrating evidence from developmental, histopathological, molecular, and functional studies, the review highlights the translational value of zebrafish as an experimental model for understanding heavy metal-associated retinal disease, identifying biomarkers of toxicity, and supporting the development of future therapeutic strategies.

## 2. Materials and Methods

### 2.1. Identification of Relevant Literature

The literature for this review was identified through a comprehensive search of the scientific publications addressing heavy metal-induced ocular and retinal toxicity in zebrafish (*Danio rerio*). Electronic searches were conducted using PubMed, Web of Science, Scopus, and ScienceDirect, covering studies published from 2003 to June 2026. This search period was selected to include both foundational research of zebrafish retinal biology and recent investigations into environmental contaminants and visual system toxicity.

Electronic searches were conducted using PubMed, Web of Science, Scopus, and ScienceDirect, covering studies published from 2003 to June 2026. The year 2003 was selected a priori as a pragmatic lower boundary for the modern zebrafish ocular toxicology literature, allowing the review to focus on studies using contemporary experimental approaches to retinal development, visual function, and environmental toxicology. Studies published before 2003 were not systematically included in the database search; however, reference lists of relevant articles, reviews, and book chapters were manually screened to identify earlier publications of particular relevance to the biological and methodological background of the review. June 2026 corresponded to the final literature search conducted during manuscript preparation.

Search terms were selected to address three principal domains: (i) zebrafish biology, (ii) ocular and retinal endpoints, and (iii) heavy metal exposure. Keywords included “zebrafish”, “Danio rerio”, “retina”, “eye”, “visual system”, “retinal degeneration”, “photoreceptor”, “retinal pigment epithelium”, “ocular toxicity”, “cadmium”, “lead”, “arsenic”, “chromium”, “copper”, “mercury”, “heavy metals”, “oxidative stress”, “apoptosis”, “neuroinflammation”, “retinal regeneration”, “optomotor response”, and “optokinetic reflex”. Boolean operators and database-specific search filters were used to enhance the retrieval of relevant publications.

To maximize evidence coverage, reference lists of relevant articles, reviews, and book chapters were also examined manually. The search strategy aimed to ensure comprehensive inclusion of studies addressing retinal morphology, visual function, molecular mechanisms of toxicity, regenerative responses, and behavioral outcomes associated with heavy metal exposure.

**Figure 1:**
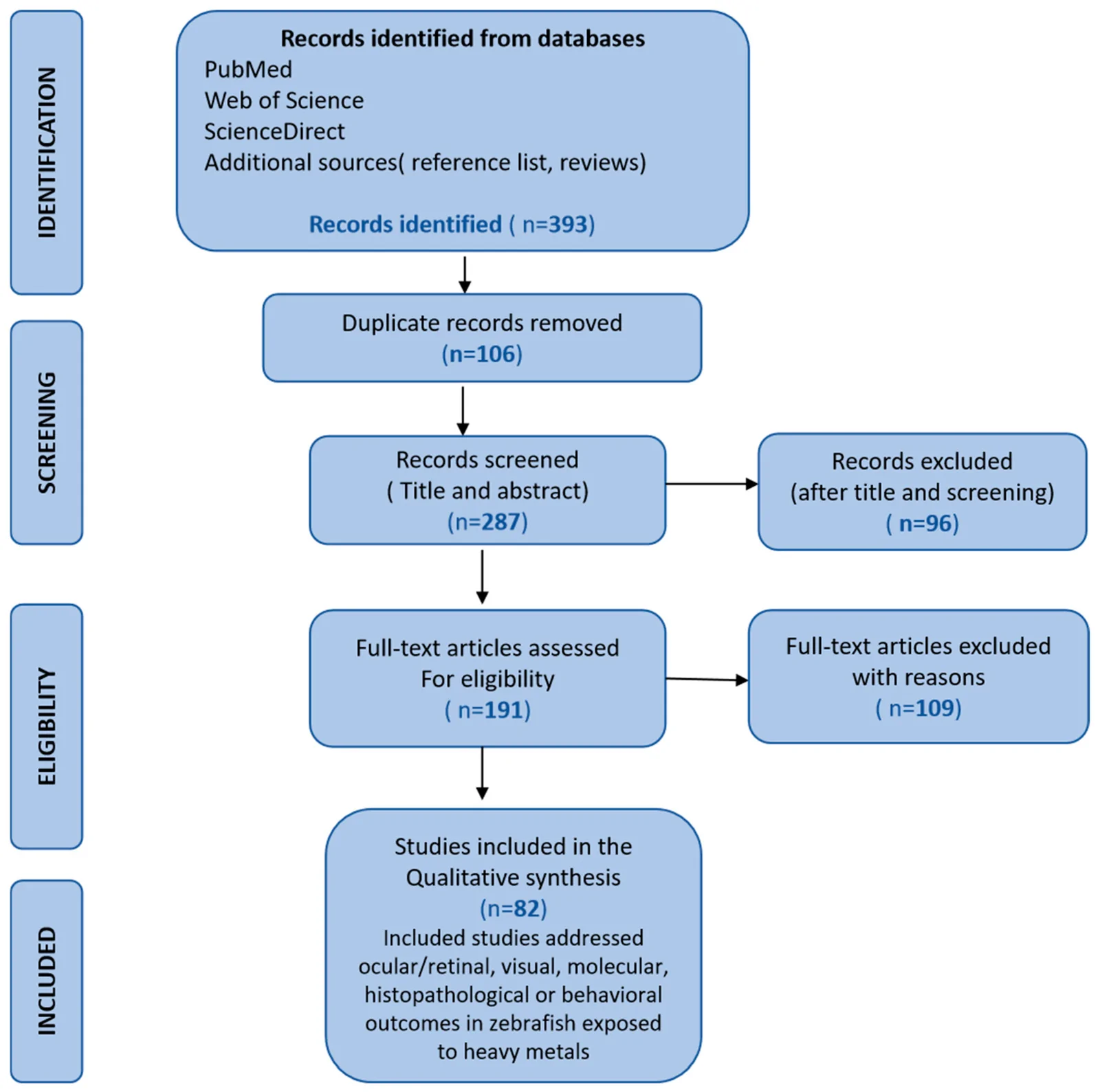
PRISMA-inspired flow diagram of the literature search and study selection process [30]. Following duplicate removal, title/abstract screening, and full-text eligibility assessment, 82 studies were included in the final qualitative synthesis.

A total of 393 records were identified through electronic databases and supplementary sources. After duplicate publications were removed, 287 records remained for title and abstract screening. Following initial screening, 191 articles were selected for full-text assessment. Studies were excluded if they did not address retinal, ocular, or visually mediated outcomes, lacked experimental data, or did not involve zebrafish. Ultimately, 82 studies were included in the final qualitative synthesis (Figure 1).

### 2.2. Eligibility Criteria and Study Selection

Studies were included in the present review if they fulfilled the following criteria:

- Experimental investigations involving zebrafish (*Danio rerio*);
- Exposure to heavy metals or metalloids, such as cadmium, lead, arsenic, chromium, copper, mercury, or environmentally relevant metal mixtures;
- Evaluation of ocular, retinal, visual, developmental, molecular, histopathological, regenerative, or behavioral endpoints associated with the visual system;
- Publication in peer-reviewed scientific journals and written in English.

Studies were excluded if they:

- Focused exclusively on environmental monitoring without biological endpoints;
- Investigated non-zebrafish species;
- Examined systemic toxicity without reporting ocular or visual system outcomes;
- Lacked primary experimental data.

Studies involving both embryonic/larval and adult zebrafish were considered. In addition to in vivo experiments, mechanistic investigations of retinal cells, molecular analyses, transcriptomics, proteomics, metabolomics, and regenerative studies were included when relevant to heavy metal-induced ocular toxicity.

### 2.3. Data Collection and Narrative Analysis

Data from eligible studies were systematically compiled and categorized based on the type of metal investigated, exposure conditions, developmental stage, analytical methodology, and reported ocular outcomes. Emphasis was given to retinal histopathology, photoreceptor integrity, alterations in the retinal pigment epithelium, oxidative stress responses, apoptosis, neuroinflammation, visual behavior, and regenerative processes.

When available, additional data concerning molecular mechanisms, such as antioxidant signaling pathways, inflammatory mediators, cell-death regulators, developmental genes, and regeneration-associated factors, were recorded. Studies employing transcriptomic, proteomic, metabolomic, or other systems-biology approaches were evaluated separately to identify emerging mechanistic insights and potential biomarkers of toxicity.

Due to the heterogeneity of exposure paradigms, experimental designs, endpoints, and analytical methodologies across the available studies, a quantitative meta-analysis was not feasible. Therefore, findings were synthesized narratively and organized into thematic sections covering retinal anatomy and physiology, mechanisms of retinal injury, metal-specific toxicological effects, mixture toxicity, visually mediated behavioral assays, retinal regeneration, and modern molecular approaches used in ocular toxicology research.

This strategy enabled the identification of recurring pathological patterns, shared mechanistic pathways, and key areas requiring further investigation in the context of heavy metal-induced retinal toxicity.

## 3. Ocular Anatomy and Conserved Retinal Features in Zebrafish (*Danio rerio*)

The zebrafish (*Danio rerio*) is now recognized as one of the most widely used vertebrate models in ophthalmological research due to its experimental accessibility, genetic tractability, and significant anatomical and functional similarities to the human visual system. The zebrafish eye possesses the main structural components of the vertebrate visual system, such as the cornea, lens, retina, retinal pigment epithelium (RPE), choroid, sclera, and optic nerve. This structural similarity provides a robust platform for investigating retinal development, visual function, ocular disease, and environmentally induced retinal toxicity [10,11,13,31]. In addition to ophthalmological research, zebrafish serve as an established model for investigating the neurotoxic effects of various environmental contaminants. This versatility further underscores their value for studying conserved mechanisms of environmentally induced neural and visual system dysfunction [12]. The high degree of conservation of retinal organization, visual signaling pathways, and developmental processes has contributed to the widespread use of zebrafish for studying the ocular effects of environmental contaminants, particularly heavy metals [7,8]. Table 1 summarizes the principal anatomical, physiological, and experimental characteristics that support the use of zebrafish as a model for ocular toxicology.

The retina is the primary target of heavy metal-induced ocular toxicity and is among the most metabolically active tissues in vertebrates. Due to its high oxygen consumption, substantial mitochondrial content, continuous exposure to light, and elevated concentration of polyunsaturated fatty acids, the retina is particularly susceptible to oxidative stress and toxicant-induced injury [5,6,7,8]. Histological analysis reveals that the zebrafish retina displays the characteristic laminar organization conserved across vertebrates, comprising three nuclear layers separated by two synaptic plexiform layers. These include the outer nuclear layer (ONL), which contains photoreceptor nuclei; the outer plexiform layer (OPL), where photoreceptors synapse with bipolar and horizontal cells; the inner nuclear layer (INL), which contains bipolar, horizontal, amacrine, and Müller glial cells; the inner plexiform layer (IPL); and the ganglion cell layer (GCL), which contains retinal ganglion cells whose axons converge to form the optic nerve [10,13]. The precise organization of these retinal layers is critical for normal visual signal processing and provides a sensitive structural framework for assessing retinal injury following heavy metal exposure.

A defining feature of the zebrafish visual system is its cone-rich retina. In contrast to nocturnal rodent models, which rely predominantly on rod-mediated vision, zebrafish are diurnal and possess four morphologically and functionally distinct cone photoreceptor subtypes. These are sensitive to ultraviolet (UV), short (S), medium (M), and long (L) wavelengths, which enables tetrachromatic color vision [10,14,15]. This cone-dominant retinal organization more closely resembles the human photopic visual system and enhances the translational value of zebrafish for investigating photoreceptor degeneration, retinal neurotoxicity, color vision deficits, and environmentally induced visual dysfunction.

Although zebrafish lack a true macula and fovea, specialized retinal regions with increased cone density and enhanced visual acuity have been identified and are considered functional analogs of the human central retina [16,17]. Additionally, approximately 70% of human genes have at least one zebrafish ortholog, including many genes involved in retinal development, phototransduction, visual processing, and inherited retinal disorders [10,11]. These conserved anatomical and genetic features underscore the translational relevance of zebrafish as a model for investigating both developmental and acquired retinal diseases.

The retinal pigment epithelium (RPE) is essential to retinal homeostasis and represents an important target of environmental toxicants. This monolayer of pigmented epithelial cells supports photoreceptor survival by mediating nutrient transport, sustaining the visual cycle, phagocytosing shed photoreceptor outer segments, and providing protection against oxidative stress. Therefore, damage to the RPE induced by toxicants may result in secondary photoreceptor degeneration, retinal dysfunction, and progressive vision loss [7,8].

In contrast to mammals, zebrafish exhibit an exceptional capacity for retinal regeneration. Müller glial cells, which extend across the entire thickness of the retina, maintain retinal homeostasis through structural support, metabolic regulation, neurotransmitter recycling, and ion balance under physiological conditions. Following retinal injury, these cells dedifferentiate, re-enter the cell cycle, and generate retinal progenitor cells capable of replacing damaged photoreceptors, interneurons, and retinal ganglion cells [18,19,20,21,22]. This regenerative response represents a key advantage of zebrafish as a model for investigating both retinal degeneration and endogenous repair mechanisms after toxicant exposure.

The rapid development of the zebrafish visual system further enhances its utility in ocular toxicology research. Eye formation begins within the first 24 h post-fertilization, and functional vision is established by approximately 4–5 days post-fertilization [10,15,17]. In combination with the optical transparency of embryos and larvae, this developmental timeline enables direct visualization of retinal morphogenesis and facilitates early detection of developmental abnormalities caused by environmental contaminants. Therefore, zebrafish provide a powerful experimental platform for investigating the effects of heavy metals on retinal development, retinal architecture, visual function, and regenerative responses throughout the life cycle.

## 4. Molecular Mechanisms of Heavy Metal-Induced Retinal Toxicity

Heavy metal-induced retinal toxicity is a multifactorial process involving interconnected molecular and cellular pathways that ultimately disrupt retinal homeostasis and visual function. While individual metals possess distinct toxicological profiles, multiple studies have demonstrated that oxidative stress, mitochondrial dysfunction, endoplasmic reticulum (ER) stress, apoptosis, neuroinflammation, and impaired retinal development are common mechanisms underlying retinal injury after exposure to cadmium (Cd), lead (Pb), arsenic (As), chromium (Cr), copper (Cu), mercury (Hg), and metal mixtures [1,6,7,8]. These pathways frequently interact and amplify one another, leading to progressive retinal degeneration, impaired visual function, and long-term neurodevelopmental consequences.

### 4.1. Oxidative Stress and Reactive Oxygen Species

Oxidative stress is widely recognized as a principal mechanism responsible for heavy metal-induced retinal injury. Due to its distinct metabolic and structural characteristics, the retina is highly susceptible to oxidative damage given its elevated oxygen consumption, high mitochondrial density, abundance of polyunsaturated fatty acids, and continuous exposure to photo-oxidative stress [5,6,7,8]. Under normal physiological conditions, reactive oxygen species (ROS) are tightly regulated by endogenous antioxidant systems. However, heavy metals can disrupt this balance by promoting ROS production and impairing antioxidant defenses [7,32].

Experimental studies in zebrafish have shown increased ROS generation after exposure to cadmium, arsenic, lead, copper, and chromium [28,29,33,34,35]. Excessive ROS production induces lipid peroxidation, protein oxidation, mitochondrial dysfunction, DNA damage, and disrupts cellular homeostasis. Additionally, alterations in antioxidant enzymes, such as superoxide dismutase (SOD), catalase (CAT), glutathione peroxidase (GPx), and components of the nuclear factor erythroid 2-related factor 2/heme oxygenase-1 (Nrf2/HO-1) signaling pathway, have been reported following metal exposure [33,34,36]. Chronic oxidative stress ultimately leads to photoreceptor degeneration, retinal thinning, and visual dysfunction, representing a shared pathogenic mechanism among various heavy metals [7,8,27].

### 4.2. Mitochondrial Dysfunction

Mitochondria play a critical role in retinal physiology by providing the substantial energy required for phototransduction, neurotransmission, and the maintenance of ionic gradients. Photoreceptors and retinal pigment epithelial cells contain exceptionally high mitochondrial densities, rendering them especially vulnerable to mitochondrial injury [8]. Consequently, disruption of mitochondrial function constitutes a key mechanism of heavy metal-induced retinal toxicity.

Heavy metals can impair mitochondrial respiration, decrease ATP synthesis, disrupt mitochondrial membrane potential, and promote excessive mitochondrial ROS production. Cadmium, arsenic, and lead have been associated with mitochondrial dysfunction through disruption of calcium homeostasis, inhibition of respiratory chain enzymes, and induction of oxidative damage [32,37,38]. Mitochondrial impairment leads to cellular energy deficits and activates intrinsic apoptotic pathways. Progressive mitochondrial damage may ultimately result in photoreceptor degeneration, retinal thinning, and impaired visual performance.

### 4.3. Endoplasmic Reticulum Stress

The endoplasmic reticulum (ER) is responsible for protein synthesis, folding, and trafficking. Disturbance of ER homeostasis leads to the accumulation of misfolded proteins and triggers the unfolded protein response (UPR), a protective mechanism that attempts to restore cellular equilibrium. Persistent ER stress, however, can initiate apoptotic signaling pathways and contribute to retinal degeneration.

Recent studies indicate that metal-induced retinal toxicity involves activation of ER stress pathways, which interact closely with oxidative stress and mitochondrial dysfunction. Copper-induced retinal injury has been linked to increased expression of ER stress-related markers, elevated ROS production, and retinal apoptosis [39]. Similarly, cadmium and lead exposure have been associated with developmental toxicity mediated by ER stress-related cellular responses [37,40]. Although the contribution of ER stress to retinal pathology has been less extensively investigated compared to oxidative stress, current evidence indicates that ER stress is a significant mediator of heavy metal-induced retinal injury.

### 4.4. Apoptosis and Emerging Cell Death Pathways

Apoptosis is among the most frequently documented cell death (RCD) mechanisms associated with heavy metal exposure in zebrafish retinal tissues. Histopathological and molecular studies have demonstrated increased apoptotic activity following exposure to cadmium, arsenic, chromium, copper, and pollutant mixtures [24,25,28,29,36]. Apoptotic degeneration is characterized by chromatin condensation, nuclear pyknosis, DNA fragmentation, and activation of caspase-dependent signaling pathways.

Heavy metal-induced apoptosis is closely interconnected with oxidative stress, mitochondrial dysfunction, endoplasmic reticulum stress, and DNA damage. Accordingly, alterations in pro-apoptotic and anti-apoptotic mediators, including *Bax*, *Bcl-2*, *caspase-3*, and *caspase-9*, have been associated with retinal injury and developmental neurotoxicity [28,33,36]. However, increasing evidence indicates that heavy metal-induced neurotoxicity involves a considerably broader spectrum of cellular stress responses and RCD mechanisms, including macroautophagy, ferroptosis, pyroptosis, necroptosis, and cuproptosis.

Macroautophagy is a lysosome-dependent degradation and recycling process that contributes to cellular homeostasis through the removal of damaged proteins and organelles. Under moderate cellular stress, activation of autophagic flux may exert a cytoprotective effect by limiting the accumulation of oxidized macromolecules and dysfunctional mitochondria. Conversely, persistent metal-induced oxidative stress, mitochondrial dysfunction, and endoplasmic reticulum stress may disrupt autophagic homeostasis and contribute to cellular injury. Selective elimination of damaged mitochondria through mitophagy is particularly relevant to metal-induced neurotoxicity because mitochondrial impairment represents a recurrent consequence of heavy metal exposure. Recent evidence from combined exposure to lead and polystyrene nanoplastics demonstrated disruption of the mitochondrial fission–mitophagy axis and associated neuronal injury, further supporting a relationship between metal-associated toxicity, mitochondrial quality control, and neurotoxicity [41]. Nevertheless, macroautophagy and mitophagy have not yet been comprehensively characterized in the heavy metal-exposed zebrafish retina, and their specific roles in retinal injury remain to be established.

Ferroptosis is an iron-dependent form of RCD characterized by excessive lipid peroxidation, disruption of redox homeostasis, and failure of cellular antioxidant defenses [42]. This pathway is particularly relevant to retinal tissue because of its high oxygen consumption, abundance of polyunsaturated fatty acids, and susceptibility to oxidative membrane damage. Heavy metal-induced ROS generation, mitochondrial dysfunction, and lipid peroxidation therefore provide conditions potentially favorable to ferroptotic injury. Recent studies have strengthened the association between metal exposure and regulated necrotic cell death pathways. In particular, a 2026 study demonstrated that cadmium-induced neurotoxicity involves mitochondrial ROS-dependent activation of the receptor-interacting protein kinase 1/receptor-interacting protein kinase 3–mixed lineage kinase domain-like protein (RIPK1/RIPK3–MLKL) pathway, indicating the involvement of necroptosis [43,44]. Although these findings broaden the spectrum of RCD mechanisms associated with metal neurotoxicity, direct characterization of ferroptosis and necroptosis within specific zebrafish retinal cell populations remains limited.

Pyroptosis represents an inflammatory form of RCD that is mechanistically linked to inflammasome activation, inflammatory caspase signaling, gasdermin-mediated membrane permeabilization, and the release of pro-inflammatory mediators. This pathway is particularly relevant to heavy metal neurotoxicity because oxidative stress and mitochondrial dysfunction can promote inflammasome-associated signaling, thereby connecting cellular damage with neuroinflammation. Recent experimental evidence indicates that copper-induced neurotoxicity can involve activation of the thioredoxin-interacting protein/transient receptor potential vanilloid 1–NLR family pyrin domain containing 3 (TXNIP/TRPV1–NLRP3) inflammatory axis and caspase-1/gasdermin D (GSDMD)-mediated pyroptosis [45]. These findings support an interaction among copper dyshomeostasis, oxidative stress, inflammatory signaling, and RCD. However, direct evidence of heavy metal-induced pyroptosis in the zebrafish retina remains scarce, and this pathway should therefore be considered an emerging mechanism requiring retinal-specific validation.

Cuproptosis is a recently characterized copper-dependent form of RCD that is mechanistically distinct from apoptosis, ferroptosis, and pyroptosis. Excess intracellular copper interacts with lipoylated proteins of the mitochondrial tricarboxylic acid cycle, promoting protein aggregation, destabilization of iron–sulfur cluster proteins, proteotoxic stress, and mitochondrial dysfunction. Recent experimental evidence indicates that inhibition of cuproptosis can attenuate copper-induced mitochondrial damage and neuronal loss, further supporting a relationship between copper dyshomeostasis, cuproptosis, and neurotoxicity [46]. Contemporary literature also identifies altered copper homeostasis and cuproptosis as emerging mechanisms in neurological disorders [47]. Given that copper exposure in zebrafish has already been associated with ROS generation, endoplasmic reticulum stress, apoptosis, and impaired retinal development [39], cuproptosis represents a biologically plausible additional mechanism of copper-induced retinal injury. Nevertheless, direct evidence of cuproptosis in the zebrafish retina is currently lacking, and its involvement should therefore be regarded as an important hypothesis for future investigation rather than an established mechanism of retinal toxicity.

Collectively, current evidence supports a broader interpretation of heavy metal-induced retinal injury in which apoptosis represents only one component of an interconnected network of cellular stress and RCD pathways. Macroautophagy and mitophagy may initially contribute to cellular adaptation and organelle quality control, whereas persistent oxidative, mitochondrial, and inflammatory stress may favor apoptosis, ferroptosis, pyroptosis, necroptosis, or, in the context of copper dyshomeostasis, cuproptosis. A new type of cell death that was discovered in recent years is ferroptosis, which is usually accompanied by a large amount of iron accumulation and lipid peroxidation during the cell death process [48]. The relative contribution and potential crosstalk among these mechanisms are likely to depend on the metal involved, exposure concentration and duration, developmental stage, and retinal cell type. Future studies combining pathway-specific molecular markers with histopathological and functional visual endpoints will be necessary to establish the contribution of these RCD programs to heavy metal-induced injury in photoreceptors, retinal ganglion cells, Müller glia, and retinal pigment epithelial cells.

### 4.5. Neuroinflammation and Developmental Neurotoxicity

Heavy metals induce retinal injury both through direct cellular toxicity and by activating inflammatory and neurodevelopmental pathways. As the retina constitutes an extension of the central nervous system, inflammatory responses within retinal tissues may significantly contribute to neuronal dysfunction and degeneration.

Experimental studies have demonstrated microglial activation, altered inflammatory signaling, and dysregulation of genes involved in neural development following cadmium exposure [1,49]. Furthermore, oxidative stress-induced inflammatory responses have been reported after combined toxicant exposure [35]. Exposure to lead and arsenic has also been associated with behavioral abnormalities, impaired neurodevelopment, and disruption of visual processing pathways [23,50]. Chronic neuroinflammation may exacerbate oxidative stress, promote neuronal death, and contribute to progressive visual impairment.

### 4.6. Impaired Retinogenesis and Ocular Development

Developing retinal tissues are highly vulnerable to environmental toxicants, such as heavy metals, because they rely on precisely regulated signaling pathways during retinal differentiation and ocular morphogenesis [24,26].

Developmental exposure to cadmium is associated with microphthalmia, retinal disorganization, impaired photoreceptor differentiation, and defective optic nerve formation [24,26]. Similarly, arsenic exposure alters the expression of key developmental regulators including *pax6a*, *pax2a*, *sox2*, and *ascl1a*, leading to abnormalities of retinal architecture and visual system development [28,51,52,53].

**Figure 2:**
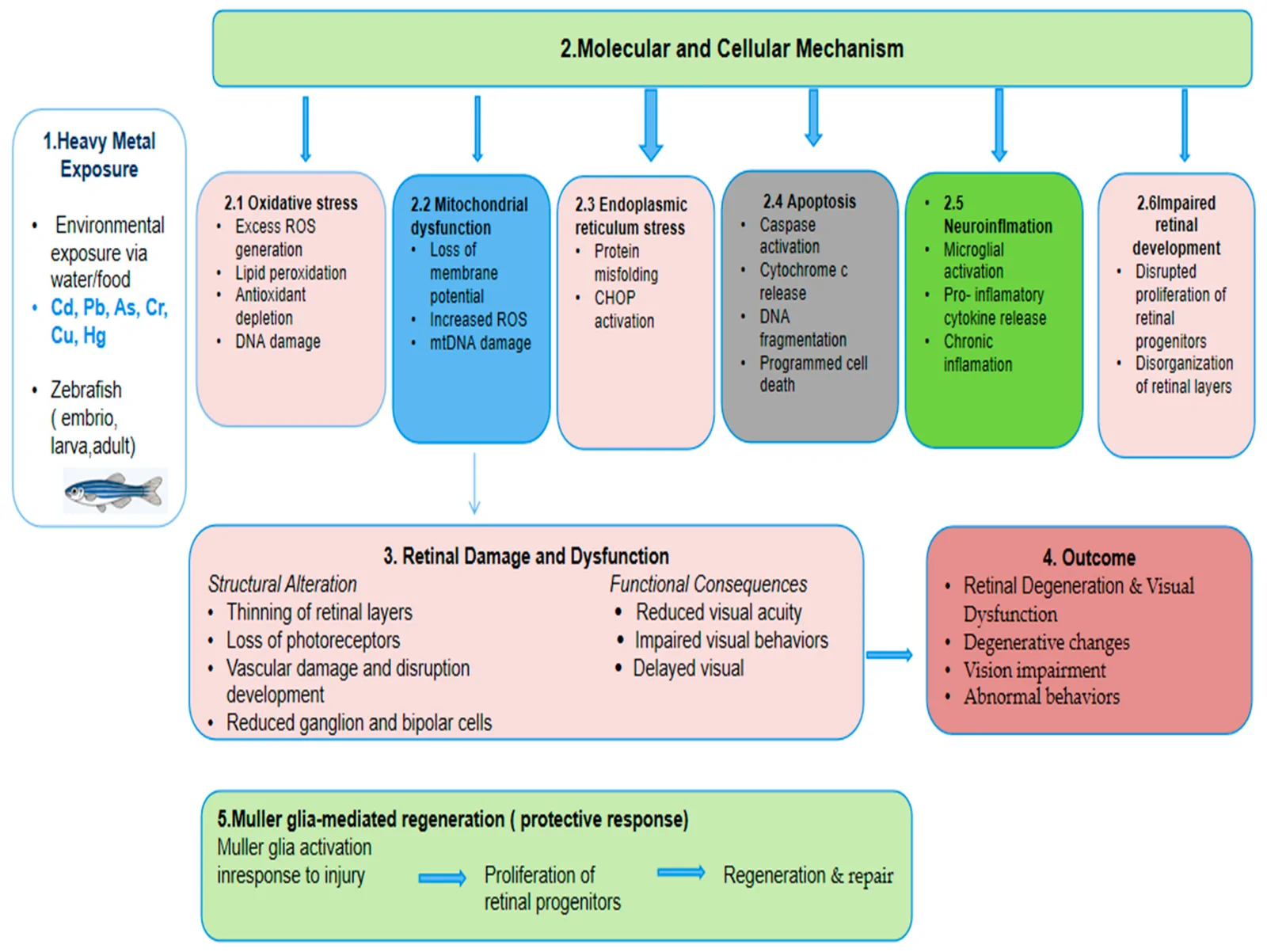
**Molecular mechanisms underlying heavy metal-induced retinal toxicity in zebrafish.** Heavy metal exposure promotes oxidative stress, mitochondrial dysfunction, endoplasmic reticulum stress, altered autophagy/mitophagy, neuroinflammation, and disruption of retinal development. In addition to apoptosis, emerging evidence implicates other regulated cell death pathways, including ferroptosis, pyroptosis, necroptosis, and cuproptosis, although their direct involvement in the zebrafish retina remains incompletely characterized. These interconnected processes may ultimately contribute to retinal degeneration and visual dysfunction. The schematic was synthesized from evidence reported by [1,7,8,28,29,33,34,36,38,40,41,43,44,45,46,47]. Abreviations: ROS, reactive oxygen species; ER, endoplasmic reticulum, RCD, regulated cell death.

Heavy metal-induced dysregulation of developmental signaling pathways can produce long-lasting consequences that extend beyond the exposure period. Disrupted retinogenesis impairs retinal connectivity, visual processing, and visually mediated behaviors, emphasizing the significance of developmental exposure studies for understanding environmentally induced visual dysfunction (Figure 2).

## 5. Histopathological and Functional Alterations Induced by Heavy Metals in the Zebrafish Retina

Heavy metals are among the most extensively investigated environmental contaminants impacting ocular development and retinal integrity in zebrafish. Although these metals differ in physicochemical properties, bioaccumulation patterns, and molecular targets, exposure frequently leads to retinal degeneration, disruption of retinal architecture, photoreceptor injury, retinal pigment epithelium (RPE) damage, and impaired visual function. The severity and characteristics of these alterations are influenced by several factors, including developmental stage, exposure duration, concentration, and the specific metal involved. Overall, current evidence indicates that the zebrafish retina is highly sensitive to metal-induced toxicity and provides a valuable model for investigating the mechanisms of environmentally induced visual dysfunction [6,7,8].

### 5.1. Cadmium (Cd)

Cadmium is one of the most extensively studied retinotoxic metals in zebrafish and consistently causes severe structural, molecular, and functional alterations in both developing and adult visual systems. Its long biological half-life and strong bioaccumulative properties make cadmium a significant environmental and public health concern [37,38].

Developmental exposure to cadmium is associated with profound ocular abnormalities, such as microphthalmia, delayed eye development, retinal disorganization, reduced retinal thickness, and optic nerve defects [24,26]. Histopathological analyses revealed extensive vacuolization across multiple retinal layers, particularly the ganglion cell layer and nerve fiber layer, along with disruption of normal retinal lamination. Further studies report degeneration of photoreceptor cells, altered retinal neuronal organization, and impaired retinal maturation following chronic exposure [25,49].

At the molecular level, cadmium induces oxidative stress, mitochondrial dysfunction, endoplasmic reticulum stress, and apoptosis. Increased production of reactive oxygen species, lipid peroxidation, and disruption of antioxidant defense systems have been documented following cadmium exposure, supporting oxidative stress as a central mechanism of retinal injury [36,54,55]. Altered expression of cone opsins and developmental regulators further suggests that cadmium interferes with retinal maturation during critical developmental periods [26].

Functional consequences of cadmium exposure include impaired visually mediated behaviors, altered locomotor activity, and reduced visual performance [27]. Collectively, the available evidence demonstrates that cadmium induces some of the most severe retinal alterations observed in zebrafish, affecting retinal development, structure, and visual function through multiple interconnected mechanisms.

### 5.2. Lead (Pb)

Lead is one of the most prevalent environmental contaminants worldwide and is recognized as a potent developmental neurotoxicant. Multiple studies have demonstrated that the visual system is a primary target of lead toxicity, especially during embryonic and larval development [23,33,34].

Developmental exposure to lead has been associated with retinal degeneration, lens abnormalities, and alterations in the retinal pigment epithelium at environmentally relevant concentrations [23]. Compared with cadmium, lead generally causes less extensive retinal destruction but has significant effects on retinal development, neuronal differentiation, and visual function.

Mechanistically, lead-induced retinal toxicity has been associated with oxidative stress, disrupted calcium homeostasis, mitochondrial dysfunction, and interference with neuronal signaling pathways [33,34,40]. Since calcium signaling is critical for retinal development and neurotransmission, disturbances in calcium-dependent processes likely contribute to the visual deficits observed after exposure.

Behavioral studies have shown impaired optomotor responses, altered startle responses, and deficits in visually guided behaviors, indicating disruption of retinal processing and visual pathway integrity [23,27].

### 5.3. Arsenic (As)

Arsenic, a naturally occurring metalloid, contaminates drinking water sources and aquatic ecosystems worldwide. While research has traditionally focused on its systemic toxicity, recent evidence indicates that arsenic also significantly impairs retinal development and visual function.

Unlike cadmium, which primarily induces severe retinal degeneration, arsenic exposure frequently results in abnormalities related to ocular development and retinal neurogenesis. Developmental exposure has been linked to altered eye morphology, retinal disorganization, thinning of the retinal pigment epithelium, and abnormal ocular enlargement during embryogenesis [28,53].

Molecular investigations have identified dysregulation of genes involved in retinal differentiation and neurodevelopment, such as *ascl1a*, *pax6a*, *pax2a*, and *sox2*. Arsenic-induced oxidative stress is considered a central mechanism underlying these developmental abnormalities, as it disrupts cellular proliferation, neuronal differentiation, and survival pathways [3,28].

In zebrafish, arsenic exposure has been associated with behavioral abnormalities, developmental neurotoxicity, oxidative stress, and disruption of visual system development [43,48,49,50].

### 5.4. Chromium (Cr)

Chromium exists in several oxidation states, among which hexavalent chromium [Cr(VI)] is of particular toxicological concern because of its strong oxidative properties and environmental relevance. In zebrafish, the available evidence directly addressing chromium-induced ocular toxicity has primarily focused on Cr(VI). Developmental exposure has been associated with reduced ocular dimensions and structural abnormalities of the retina, including retinal cell nuclear pyknosis and increased separation between the lens and retina [29]. These alterations were accompanied by increased reactive oxygen species (ROS) generation and apoptosis, supporting oxidative stress-mediated cellular injury as an important mechanism of Cr(VI)-induced ocular toxicity. At the molecular level, Cr(VI) exposure affected genes involved in antioxidant defense and apoptosis and altered the expression of genes associated with specific retinal cell populations, crystallins, and opsins [29]. Photoreceptor-associated genes were particularly affected, indicating that chromium exposure may interfere with retinal differentiation and photoreceptor development in addition to producing direct cellular injury. Taken together, the available findings indicate that chromium, particularly in its hexavalent form, can disrupt zebrafish eye development through interconnected oxidative, apoptotic, and developmental mechanisms [29].

### 5.5. Copper (Cu)

Copper is an essential trace element involved in various physiological processes, such as mitochondrial respiration, antioxidant defense, and neurotransmitter synthesis. Disruption of copper homeostasis can lead to substantial retinal toxicity.

Experimental studies have demonstrated that excessive copper exposure induces retinal degeneration, which is characterized by reduced retinal diameter, decreased ganglion cell density, photoreceptor injury, and increased apoptosis [39]. These histopathological alterations are frequently accompanied by disorganization of retinal layers and reduced cellularity across multiple retinal compartments.

Mechanistic investigations have identified oxidative stress and endoplasmic reticulum stress as major contributors to copper-induced retinal injury. Elevated ROS generation, mitochondrial dysfunction, and activation of apoptosis-related signaling pathways have been observed following exposure [35,39]. Since copper is essential for normal retinal physiology, retinal injury is primarily attributed to disruption of copper homeostasis rather than simple accumulation.

### 5.6. Mercury (Hg)

Mercury-induced ocular toxicity has been investigated less extensively in zebrafish in comparison to other metals. Mercury readily crosses biological membranes and accumulates in neural tissues, making the retina particularly vulnerable to its toxic effects. Consequently, mercury exposure can significantly impair retinal development and visual function.

Developmental exposure to methylmercury is associated with alterations in retinal physiology, deficits in visually mediated behaviors, and neurodevelopmental abnormalities. Mercury-induced toxicity primarily occurs through mechanisms such as oxidative stress, disruption of calcium homeostasis, mitochondrial dysfunction, and interference with neuronal signaling pathways [1,56,57]. The main histopathological, molecular, and functional alterations observed following exposure to individual heavy metals are summarized in Table 2.

## 6. Mixture Toxicity and Environmentally Relevant Exposure Scenarios

Environmental exposure to heavy metals seldom involves a single contaminant. Aquatic organisms are typically exposed to complex mixtures of metals derived from industrial discharges, mining activities, agricultural runoff, wastewater effluents, and urban pollution. Therefore, assessing the toxicity of individual metals in isolation may underestimate the biological effects experienced under environmentally relevant conditions. Recent zebrafish studies demonstrate that interactions among heavy metals can alter toxicological responses through additive, synergistic, or antagonistic mechanisms, leading to outcomes that differ substantially from those predicted by single-compound exposures [1,35,59].

### 6.1. Environmental Relevance of Metal Mixtures

Aquatic ecosystems often contain multiple heavy metals simultaneously, such as combinations of cadmium, lead, arsenic, copper, chromium, mercury, zinc, and nickel. These contaminants frequently target similar cellular pathways, so that co-exposure can result in cumulative toxic effects on retinal tissues and visual function. Additionally, metals can influence each other’s uptake, distribution, metabolism, and excretion, thereby altering the toxicity profiles and biological responses.

From an ecotoxicological perspective, mixture toxicity studies are particularly important because environmental concentrations of individual metals often fall below established toxicity thresholds. In contrast, combined exposure may still induce significant biological effects. Therefore, mixture-based investigations provide a more realistic assessment of environmental risk than studies that focused exclusively on single-metal exposure paradigms [1,45,52].

### 6.2. Cadmium–Lead Interactions

Cadmium and lead commonly co-occur in aquatic environments and have been extensively investigated in zebrafish mixture-toxicity studies. While both metals independently induce oxidative stress, developmental abnormalities, and neurotoxicity, their combined effects are not always predictable using simple additive models.

Heffern et al. [59] demonstrated that co-exposure to cadmium and lead significantly altered behavioral responses and circadian activity patterns in zebrafish larvae compared to exposure to either metal alone. Interestingly, both antagonistic and synergistic interactions were observed depending on the biological endpoint, indicating that the nature and magnitude of metal–metal interactions are highly context-dependent. Additional studies have reported increased oxidative damage and developmental toxicity following combined exposure, suggesting that retinal injury may be influenced by both the concentration of individual contaminants and the composition of environmental mixtures [1].

These findings highlight the complexity of metal–metal interactions and emphasize the importance of considering mixture effects when evaluating ocular and neurodevelopmental toxicity.

### 6.3. Copper–Cadmium Co-Exposure

Copper and cadmium constitute another environmentally relevant combination. Copper is an essential trace element required for mitochondrial respiration and antioxidant defense, whereas cadmium lacks any known physiological function and is highly toxic even at low concentrations.

Majid et al. [35] demonstrated that simultaneous exposure to copper and cadmium induces greater oxidative stress, DNA damage, and cellular dysfunction compared to exposure to either metal individually. At the cellular level, both metals disrupt mitochondrial function and antioxidant defense mechanisms, leading to increased generation of reactive oxygen species and impaired cellular homeostasis. These interactions may be particularly harmful to retinal tissues due to their high metabolic activity and reliance on efficient mitochondrial function.

Therefore, combined copper–cadmium exposure may increase retinal vulnerability to degeneration by amplifying oxidative and mitochondrial injury pathways. This underscores the necessity of incorporating co-exposure scenarios into environmental risk assessment.

### 6.4. Interactions with Other Environmental Contaminants

In natural environments, heavy metals rarely occur in isolation from other pollutants. Co-exposure to pesticides, pharmaceuticals, microplastics, and other environmental contaminants can further influence toxicological responses and exacerbate retinal injury.

For example, Di Paola et al. [36] demonstrated that the neonicotinoid pesticide imidacloprid intensified cadmium-induced retinal cell death in zebrafish. Combined exposure led to enhanced oxidative stress, increased apoptosis, and greater disruption of antioxidant signaling pathways, including Nrf2- and HO-1-mediated responses. These findings suggest that environmental co-contaminants can significantly amplify retinal injury compared to exposure to heavy metals alone.

These observations highlight the need to investigate complex pollutant mixtures of heavy metals and emerging contaminants that more closely reflect environmental exposure scenarios [57].

### 6.5. Developmental Consequences of Mixture Exposure

Embryonic and larval stages are highly vulnerable to contaminant mixtures. During development, heavy metals can concurrently disrupt ocular morphogenesis, retinal differentiation, neurogenesis, and photoreceptor maturation by interfering with multiple signaling pathways.

Combined exposure to heavy metals is associated with developmental abnormalities, such as delayed hatching, impaired growth, altered eye development, behavioral deficits, and increased oxidative stress [35,59]. Since retinal development relies on tightly coordinated signaling networks, simultaneous disruption of multiple pathways may lead to more severe outcomes than exposure to individual toxicants.

Developmental exposure can result in long-lasting consequences that persist into adulthood, including visual dysfunction, behavioral abnormalities, and increased susceptibility to retinal degeneration [1,36].

### 6.6. Shared Molecular Targets of Mixture Toxicity

Although different metals exhibit distinct toxicological profiles, several molecular pathways are especially vulnerable to combined metal exposure:

- Reactive oxygen species generation and oxidative stress;
- Mitochondrial dysfunction and ATP depletion;
- Calcium signaling dysregulation;
- Endoplasmic reticulum stress;
- Apoptosis and other regulated cell death pathways;
- Neuroinflammatory signaling;
- Dysregulation of developmental genes involved in retinogenesis.

As many heavy metals converge on common pathways, co-exposure can amplify cellular damage by simultaneously disrupting multiple protective mechanisms and overwhelming endogenous defense systems. Nevertheless, comparative studies demonstrate that individual metals may differ substantially in their effects on behavior, sensory processing, and neurodevelopment, even when targeting overlapping molecular pathways. This highlights the complexity of predicting toxicity outcomes for heavy metal mixtures [53,54].

Due to its high energetic demands and vulnerability to oxidative injury, the retina may represent one of the most sensitive targets for toxicity of heavy metal mixtures [7,32,35,36].

**Figure 3:**
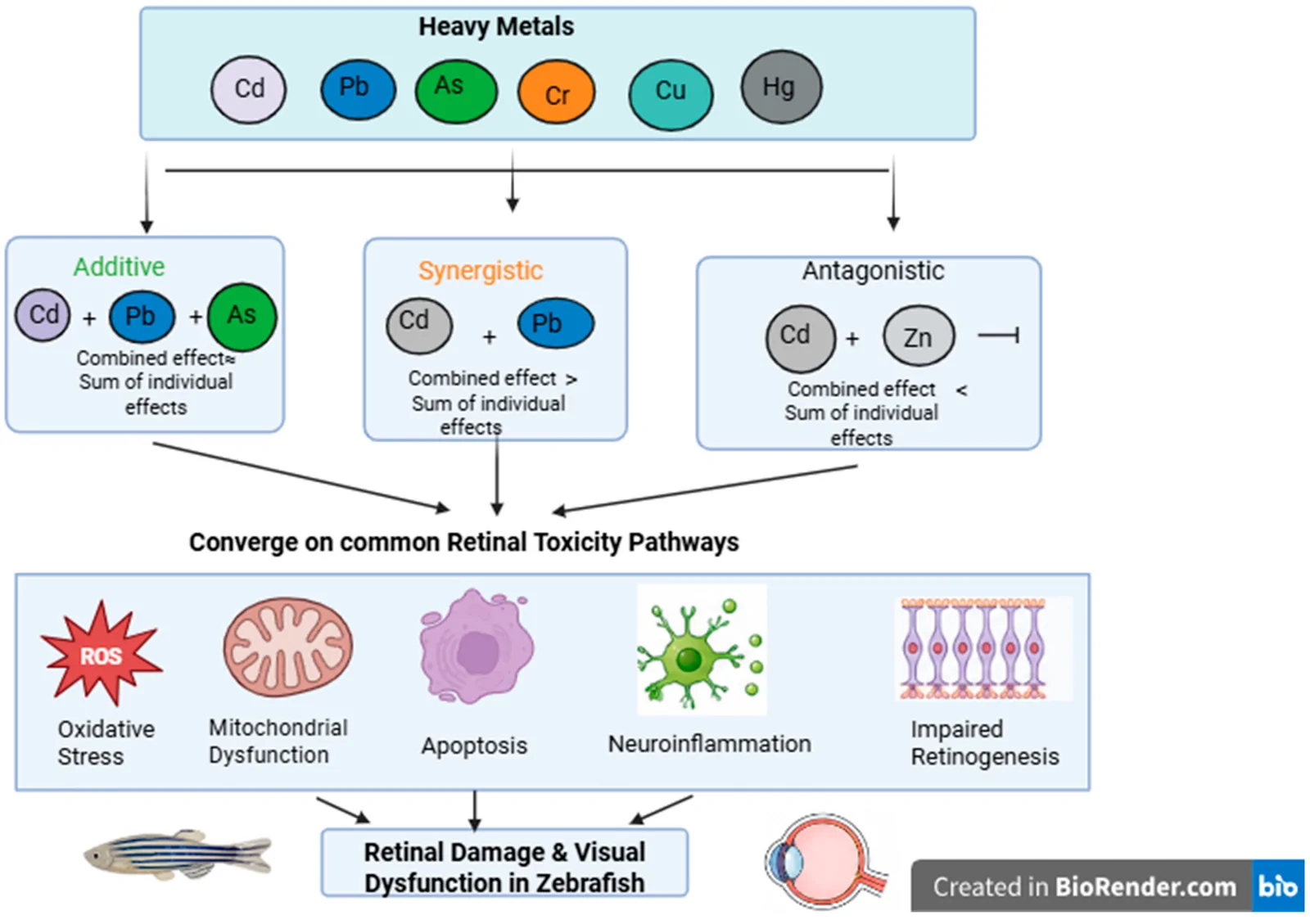
Conceptual model illustrating additive, synergistic, and antagonistic interactions among heavy metals and their convergence on common retinal toxicity pathways, including oxidative stress, mitochondrial dysfunction, apoptosis, neuroinflammation, and impaired retinogenesis. The schematic was developed based on findings reported by [1,7,8,32,35,59]. Abbreviations: Cd = cadmium; Pb = lead; As = arsenic; Cr = chromium; Cu = copper; Hg = mercury; ROS = reactive oxygen species.

Figure 3 summarizes representative examples of heavy metal mixture toxicity reported in zebrafish, including the affected biological endpoints, proposed mechanisms, and reported interactions.

### 6.7. Implications for Ocular Toxicology Research

The increasing awareness of mixture toxicity presents significant implications for future zebrafish ocular toxicology research. Most currently available studies assess individual contaminants under controlled laboratory conditions, whereas real-world environmental exposures typically involve multiple metals at variable concentrations over prolonged periods [35,59]. Future research should prioritize environmentally relevant exposure paradigms that incorporate realistic contaminant mixtures and chronic low-dose exposure scenarios. Employing integrated histopathological, behavioral, imaging, and multi-omics methodologies—including transcriptomics, proteomics, and metabolomics—may facilitate the identification of biomarkers capable of distinguishing between individual-metal toxicity and mixture-induced effects [60,61]. These approaches will improve ecological risk assessment and provide a more comprehensive understanding of the role of environmental metal contamination in retinal disease and visual dysfunction among aquatic organisms and humans [8,17] (Table 3).

## 7. Functional Assessment Through Visually Mediated Behavioral Assays

Histopathological and molecular analyses provide essential insights into retinal injury following heavy metal exposure; however, structural alterations do not always correlate directly with functional impairment. Frequently, visual dysfunction precedes overt retinal degeneration and may remain undetected by conventional histological techniques. Consequently, visually mediated behavioral assays have become indispensable tools in zebrafish ocular toxicology, providing sensitive, non-invasive, and quantifiable endpoints for evaluating the functional consequences of retinal injury [5,15,17].

The zebrafish visual system develops rapidly, with visually mediated behaviors becoming detectable within the first week of life. Because these responses rely on the integrity of photoreceptors, retinal interneurons, retinal ganglion cells, and central visual pathways, behavioral alterations may reflect disturbances occurring at multiple levels of visual processing. Additionally, behavioral assays can be performed repeatedly throughout development and adulthood, allowing longitudinal assessment of visual function following exposure to environmental contaminants. As a result, behavioral testing has become an important complement to histopathological and molecular investigations of retinal toxicity [10,15].

### 7.1. Optomotor Response (OMR)

The optomotor response (OMR) is one of the most widely used behavioral assays for assessing visual function in zebrafish. This innate behavior is triggered by exposure to moving visual patterns, typically alternating black and white stripes. Zebrafish respond by swimming in the direction of the moving stimulus in an attempt to stabilize their visual environment.

Because successful OMR performance requires intact photoreceptor function, retinal signal transmission, and visual processing pathways, impairment of this behavior is considered a highly sensitive indicator of visual dysfunction. Multiple studies have reported reduced OMR performance following exposure to heavy metals and other environmental toxicants, indicating deficits in visual acuity, retinal integrity, and neuronal processing [17,23,27]. Notably, OMR alterations may occur before histologically detectable retinal degeneration, underscoring the utility of behavioral endpoints for early detection of toxic effects.

The OMR assay offers several advantages, such as simplicity, non-invasiveness, high reproducibility, and suitability for high-throughput screening. Consequently, it remains one of the most widely employed functional endpoints in developmental toxicology, ecotoxicology, and ocular toxicology studies [15,17].

### 7.2. Optokinetic Reflex (OKR)

The optokinetic reflex (OKR) is a widely used method for evaluating visual performance in zebrafish. This assay quantifies compensatory eye movements in response to moving visual stimuli, thereby providing objective measures of visual tracking ability, contrast sensitivity, and retinal function.

In contrast to OMR, which evaluates swimming behavior, the OKR directly assesses ocular motor responses and therefore provides a more precise measure of visual processing. Larval zebrafish are especially suitable for OKR testing due to their optical transparency, which facilitates direct observation of eye movements and allows accurate quantification of visual performance [10,15].

Impairment of photoreceptor function, retinal circuitry, or visual processing pathways by heavy metals can lead to reduced OKR performance. Consequently, this assay is particularly valuable for detecting subtle visual deficits that may not be evident through morphological examination alone [10,17]. Given the close association between OKR performance and retinal integrity, the assay is frequently used to assess developmental retinal toxicity and visual dysfunction.

### 7.3. Phototaxis

Phototaxis is defined as the tendency of zebrafish larvae to orient and move in response to light stimuli. This innate behavior depends on functional photoreceptors, intact retinal circuitry, and appropriate transmission of visual information to higher neural centers.

Alterations in phototactic behavior have been reported following exposure to environmental contaminants and may indicate impaired light perception, retinal dysfunction, or disruption of visual pathway development [6,51]. Due to its rapid and non-invasive assessment, phototaxis is commonly utilized in developmental toxicity studies investigating visual system impairment.

In addition to assessing retinal function, phototaxis assays can also provide insight into broader neurodevelopmental disturbances that affect sensory processing, behavioral adaptation, and neural circuit formation.

### 7.4. Visually Evoked Startle Response

The visually evoked startle response represents another significant behavioral endpoint for assessing visual system integrity. This response consists of a rapid escape movement triggered by sudden visual stimuli and relies on coordinated interactions among retinal neurons, central visual pathways, and motor circuits.

Developmental exposure to heavy metals has been associated with altered startle responses, suggesting impairment of visual processing and sensorimotor integration [23,51]. While changes in startle behavior are not exclusively attributable to retinal dysfunction, these alterations provide valuable complementary insights into the functional consequences of environmental neurotoxicity.

When interpreted alongside histopathological and molecular findings, startle response assays facilitate a more comprehensive evaluation of visual system health and environmental toxicant exposure. Collectively, these behavioral assays provide complementary data on retinal integrity, visual processing, and neurodevelopmental function, allowing for sensitive detection of visual deficits that may precede observable histopathological alterations [6,17,27].

### 7.5. Emerging Automated Behavioral Platforms

Recent advances in digital imaging, machine vision, video tracking, and automated behavioral analysis have significantly enhanced zebrafish ocular toxicology research. Modern tracking systems can simultaneously quantify swimming velocity, movement trajectories, response latency, visual preference, stimulus-following behavior, and other parameters relevant to visual function. These technologies improve sensitivity, minimize observer bias, and facilitate high-throughput screening of environmental toxicants [60,61].

With the increasing complexity of environmental exposure scenarios, automated behavioral platforms are expected to play an increasingly important role in improving the sensitivity, scalability, and mechanistic interpretation of visual toxicity assessments [17].

### 7.6. Behavioral Assays as Early Indicators of Retinal Toxicity

A key advantage of visually mediated behavioral assays is their ability to detect functional impairment before the onset of overt structural degeneration. As retinal neurons operate within highly integrated visual networks, even subtle alterations in photoreceptor function, synaptic transmission, or neuronal signaling can produce measurable behavioral deficits [17,27]. Therefore, behavioral endpoints should be regarded as sensitive indicators of early retinal dysfunction rather than solely as complementary assessments. The integration of behavioral testing with histopathological, molecular, and omics-based approaches provides a comprehensive framework for evaluating heavy metal-induced ocular toxicity and strengthens the translational relevance of zebrafish models for human retinal disease [6,17,60,61].

In summary, visually mediated behavioral assays are essential tools for assessing the functional consequences of retinal injury in zebrafish. Their sensitivity, non-invasive nature, and suitability for longitudinal studies continue to support their widespread application in environmental ocular toxicology, developmental neurotoxicology, and translational vision research [15,17,27].

## 8. Innate Retinal Regeneration Following Heavy Metal-Induced Injury

A key advantage of the zebrafish (*Danio rerio*) model in ocular toxicology research is its remarkable capacity for retinal regeneration. Unlike mammals, where retinal neuronal loss typically leads to permanent structural damage and irreversible visual impairment, zebrafish retain the ability to regenerate virtually all retinal cell types after injury. This regenerative potential positions zebrafish as a valuable experimental model for studying both the mechanisms underlying retinal degeneration and endogenous pathways that promote retinal repair and functional recovery [18,19,20,21,22].

Heavy metal exposure frequently causes retinal injury through mechanisms such as oxidative stress, mitochondrial dysfunction, apoptosis, neuroinflammation, and disruption of retinal homeostasis [1,8,28]. In most vertebrate species, such damage would result in progressive degeneration and permanent vision loss. In contrast, zebrafish possess an intrinsic regenerative response that can partially or completely restore retinal structure and function after toxicant-induced, photochemical, mechanical, and pharmacological injury [18,19,20,21].

### 8.1. Müller Glia-Mediated Retinal Regeneration

The regenerative capacity of the zebrafish retina is primarily mediated by Müller glial cells. Under physiological conditions, Müller glia preserve retinal homeostasis through structural support, metabolic regulation, neurotransmitter recycling, ion balance, and neuroprotective functions. Following retinal injury, however, these cells undergo extensive molecular and phenotypic reprogramming [18,20].

Damage-induced signaling stimulates Müller glia to dedifferentiate and re-enter the cell cycle, generating populations of proliferative retinal progenitor cells. These progenitors subsequently migrate toward damaged retinal regions and differentiate into various neuronal subtypes, such as photoreceptors, bipolar cells, amacrine cells, horizontal cells, and retinal ganglion cells [18,19,21,22]. This regenerative process enables structural reconstruction and functional restoration of damaged retinal tissues.

Because heavy metals often target photoreceptors and retinal neurons, Müller glial-mediated regeneration constitutes a crucial endogenous mechanism capable of limiting the long-term consequences of retinal injury in zebrafish.

### 8.2. Molecular Regulation of Retinal Regeneration

A complex network of transcription factors, growth factors, and intracellular signaling pathways regulates retinal regeneration. Epigenetic remodeling also plays a significant role in regenerative activation, as DNA methylation changes occurring in Müller glia facilitate partial cellular reprogramming and acquisition of progenitor-like characteristics during retinal regeneration [61].

*Ascl1a*, *lin28*, and *pax6a* are among the most extensively studied regulators. The transcription factor *ascl1a* is rapidly induced following retinal injury and plays a central role in initiating Müller glia dedifferentiation and activation of regenerative gene networks. Lin28 contributes to cellular reprogramming and expansion of progenitor populations, whereas *pax6a* regulates neuronal differentiation and retinal development [18,21,22,61].

Multiple signaling pathways further modulate regenerative responses. Notch signaling maintains Müller glia in a quiescent state under physiological conditions but is dynamically regulated following injury. Wnt/β-catenin signaling promotes progenitor proliferation and retinal repair, while Jak/STAT signaling contributes to injury-induced cellular reprogramming and regenerative activation. Fibroblast growth factor (FGF) signaling additionally supports proliferation, migration, and differentiation of retinal progenitor cells [18,19,20].

Recent studies have demonstrated that epigenetic remodeling contributes to Müller glia reprogramming and regenerative competence. Changes in DNA methylation patterns and chromatin accessibility facilitate the activation of regeneration-associated genes and support the transition from differentiated Müller glia to multipotent progenitor cells [61].

The coordinated interaction of these pathways enables zebrafish to exhibit one of the most robust regenerative responses among vertebrates.

### 8.3. Structural and Functional Recovery Following Retinal Injury

Multiple experimental studies have demonstrated substantial structural recovery following severe retinal injury in zebrafish. Regeneration has been observed after photoreceptor ablation, exposure to intense light, mechanical injury, neurotoxic damage, and chemically induced retinal degeneration [19,20,22].

Histologically, regeneration is characterized by restoration of retinal lamination, repopulation of depleted retinal layers, recovery of photoreceptor populations, and re-establishment of overall retinal architecture. In numerous experimental models, regenerated retinas are nearly indistinguishable from uninjured tissue within several weeks of the initial injury.

Importantly, retinal regeneration extends beyond structural repair to include functional recovery of visual performance. Restoration of optomotor responses, optokinetic reflexes, and other visually mediated behaviors has been reported, indicating successful integration of newly generated neurons into existing retinal circuitry. These findings suggest that regenerated retinal cells contribute meaningfully to visual processing and behavioral function rather than solely replacing lost tissue [19,21].

### 8.4. Retinal Regeneration Following Heavy Metal-Induced Injury

While most retinal regeneration studies have employed mechanical, photochemical, or genetic injury models, accumulating evidence indicates that similar regenerative mechanisms are also activated following toxicant-induced retinal damage [18,19,20]. Heavy metal exposure frequently induces photoreceptor degeneration, retinal apoptosis, oxidative stress, and disruption of retinal organization, each of which can activate Müller glia-mediated repair pathways [24,25,28,29].

Cadmium-induced retinal injury serves as a prominent example. Histopathological studies have documented retinal degeneration accompanied by evidence of proliferative and regenerative responses after toxic exposure [25]. Similar regenerative activation may also occur following exposure to arsenic, copper, chromium, and other heavy metals. However, studies specifically investigating regeneration after metal-induced retinal injury remain relatively limited [28,29,39].

It is important to recognize that heavy metals may exert dual effects on retinal tissues. Although they can activate regenerative signaling in response to tissue injury, they may also impair cellular pathways essential for successful repair. Oxidative stress, mitochondrial dysfunction, and inflammatory signaling can interfere with progenitor cell survival, differentiation, or integration [1,37,49]. Consequently, understanding how environmental contaminants influence regenerative pathways remains a critical area for future investigation [21,22].

### 8.5. Implications for Regenerative Medicine

The regenerative capacity of the zebrafish retina has implications that extend beyond the field of environmental toxicology. Human retinal disorders, including age-related macular degeneration, retinitis pigmentosa, diabetic retinopathy, and glaucoma, are characterized by progressive neuronal loss and limited endogenous regeneration. Elucidating the molecular mechanisms underlying retinal regeneration in zebrafish may facilitate the development of innovative therapeutic approaches for human retinal disease.

Recent research has focused on strategies to induce mammalian Müller glia to acquire regenerative properties similar to those observed in zebrafish. Experimental manipulation of pathways involving Ascl1, Lin28, Wnt/β-catenin, and Jak/STAT signaling has shown potential to promote limited regenerative responses in mammalian retinas [18,21]. Despite significant remaining challenges, findings from zebrafish regeneration research continue to inform the development of regenerative therapies for vision restoration.

Overall, the ability of zebrafish to regenerate retinal tissue following injury represents a major advantage of this model system. In studies of heavy metal-induced ocular toxicity, retinal regeneration provides a unique opportunity to investigate the dynamic balance between degeneration and repair, offering valuable insight into both environmental retinal pathology and future therapeutic strategies for retinal disease.

## 9. Histopathological, Immunohistochemical, and Multi-Omics Approaches for Investigating Heavy Metal-Induced Retinal Toxicity

Accurate characterization of heavy metal-induced retinal toxicity requires integrating morphological, molecular, and functional approaches. Over the past two decades, advancements in histopathology, immunohistochemistry (IHC), transcriptomics, proteomics, and metabolomics have substantially improved understanding of the mechanisms underlying retinal injury in zebrafish [6,17,60]. These methodologies provide complementary information regarding structural damage, cellular responses, molecular signaling pathways, and regenerative processes, thereby enabling comprehensive evaluation of ocular toxicity.

Heavy metal-induced retinal injury involves multiple interconnected mechanisms, such as oxidative stress, mitochondrial dysfunction, apoptosis, neuroinflammation, and impaired retinal development [1,9,28]. Therefore, integrating traditional histopathological assessment with modern molecular profiling approaches has become increasingly important. These strategies facilitate identification of biomarkers of toxicity (Table 4), improve mechanistic understanding, and provide insight into pathways involved in retinal degeneration and regeneration [18,60,61].

### 9.1. Histopathological Assessment of Retinal Injury

Histopathology remains the cornerstone of ocular toxicology research and is the primary method for assessing retinal injury following heavy metal exposure. Histological examination allows direct visualization of retinal architecture and provides detailed information regarding tissue organization, cellular integrity, and lesion severity.

In zebrafish models, retinal tissues are typically fixed in neutral buffered formalin or paraformaldehyde, embedded in paraffin, sectioned, and stained with hematoxylin and eosin (H&E). Evaluation commonly focuses on the outer nuclear layer (ONL), inner nuclear layer (INL), ganglion cell layer (GCL), photoreceptor layer, and retinal pigment epithelium (RPE). Heavy metal exposure is linked to a wide range of retinal lesions, such as microphthalmia, retinal thinning, retinal disorganization, photoreceptor degeneration, vacuolization, RPE damage, nuclear pyknosis, cellular edema, ganglion cell loss, and disruption of retinal lamination [23,24,25,26,28,29]. Of the metals investigated, cadmium generally produces the most severe histopathological alterations, including extensive vacuolization, retinal disorganization, and degeneration of multiple retinal cell populations. Consequently, histopathological analysis provides the structural framework upon which mechanistic and functional investigations are built.

### 9.2. Immunohistochemical Evaluation of Retinal Injury and Repair

Although conventional histology provides valuable insights into retinal morphology, it cannot identify specific cellular populations or molecular pathways involved in retinal injury. Immunohistochemistry (IHC) overcomes this limitation by enabling the localization and semi-quantitative assessment of proteins associated with oxidative stress, apoptosis, neuroinflammation, cellular proliferation, and retinal regeneration. As a result, IHC has become an indispensable tool for investigating the cellular and molecular mechanisms underlying heavy metal-induced retinal toxicity in zebrafish.

Apoptosis Markers

Programmed cell death represents a major pathological outcome of heavy metal exposure and is commonly assessed using immunohistochemical markers linked to apoptotic signaling pathways. Frequently used markers include activated *Caspase-3*, *Bax*, *Bcl-2*, cleaved poly(ADP-ribose) polymerase (PARP), and terminal deoxynucleotidyl transferase dUTP nick-end labeling (TUNEL). Increased Caspase-3 immunoreactivity has been reported in association with photoreceptor degeneration, retinal neuronal loss, and developmental retinal injury following exposure to environmental toxicants [28,32,36]. Evaluation of pro-apoptotic and anti-apoptotic proteins provides further insight into the balance between cell survival and cell death in injured retinal tissues.

Oxidative Stress Markers

Oxidative stress is considered a central mechanism underlying heavy metal-induced retinal toxicity; therefore, proteins involved in antioxidant defense and oxidative damage responses are frequently investigated. Commonly assessed markers include nuclear factor erythroid 2-related factor 2 (Nrf2), heme oxygenase-1 (HO-1), superoxide dismutase (SOD), catalase (CAT), 4-hydroxynonenal (4-HNE), and 8-hydroxy-2′-deoxyguanosine (8-OHdG). These biomarkers enable the evaluation of lipid peroxidation, oxidative DNA damage, and activation of endogenous antioxidant defense pathways, thereby providing valuable information about cellular responses to metal-induced oxidative injury [33,34,36].

Neuroinflammation Markers

Retinal injury is commonly accompanied by activation of glial and inflammatory responses, which contribute to neuronal dysfunction and degeneration. Consequently, immunohistochemical markers of neuroinflammation have become increasingly significant in zebrafish ocular toxicology research. Frequently used markers include glial fibrillary acidic protein (GFAP), ionized calcium-binding adaptor molecule 1 (Iba-1), tumor necrosis factor-α (TNF-α), interleukin-1β (IL-1β), and interleukin-6 (IL-6). Elevated expression of these proteins may indicate Müller glia activation, microglial recruitment, and ongoing inflammatory processes associated with retinal degeneration [1,49].

Proliferation and Regeneration Markers

The remarkable regenerative capacity of the zebrafish retina has generated significant interest in markers related to cellular proliferation and retinal repair. Frequently used indicators include proliferating cell nuclear antigen (*PCNA*), *Ki-67*, bromodeoxyuridine (*BrdU*), *Pax6*, *Ascl1a*, and *Lin28*. These markers enable identification of proliferating Müller glia, retinal progenitor cells, and regenerating neuronal populations after injury. Assessing proliferation- and regeneration-associated proteins is particularly valuable for investigating the balance between degeneration and repair following heavy metal exposure [18,20,21,22].

Collectively, immunohistochemical approaches provide a critical link between histopathological observations and molecular mechanisms of retinal toxicity. By enabling visualization of specific cellular responses within retinal tissues, IHC contributes substantially to understanding the pathways underlying retinal degeneration, neuroinflammation, oxidative injury, and regenerative processes in zebrafish exposed to heavy metals.

### 9.3. Transcriptomic Approaches

Transcriptomic technologies have significantly advanced the understanding of retinal responses to environmental contaminants. High-throughput RNA sequencing (RNA-seq), microarray analysis, and targeted gene expression profiling enable comprehensive evaluation of transcriptional alterations induced by heavy metal exposure.

Transcriptomic studies have identified multiple biological pathways disrupted by heavy metal exposure, such as oxidative stress responses, apoptotic signaling, retinogenesis, phototransduction, inflammatory signaling, mitochondrial function, and cell-cycle regulation [1,28,29]. Frequently reported genes include *pax6a*, *pax6b*, *ascl1a*, *sox2*, *rho*, *cone opsins*, *nrf2*, *bax*, and *bcl2*.

By correlating histopathological alterations with gene expression changes, transcriptomic approaches provide a critical connection between structural pathology and molecular mechanisms of toxicity. These analyses enable the identification of early transcriptional responses that may precede overt retinal degeneration and therefore represent valuable candidates for biomarker discovery.

### 9.4. Proteomic Analysis

Proteomic approaches complement transcriptomic investigations by assessing changes at the protein level. Since gene expression does not always correlate directly with protein abundance, localization, or activity, proteomic analyses provide additional insights into functional biological responses.

Mass spectrometry-based proteomic studies have identified alterations in proteins involved in energy metabolism, antioxidant defense, cytoskeletal organization, phototransduction, protein folding, and cellular stress responses. While proteomic studies specifically addressing heavy metal-induced retinal toxicity in zebrafish are currently limited, this approach demonstrates significant potential for identifying novel biomarkers and therapeutic targets related to retinal degeneration [60,61].

Proteomic analyses are particularly valuable for identifying post-translational modifications and signaling events that are not detectable through transcriptomic analyses alone.

### 9.5. Metabolomics and Emerging Multi-Omics Strategies

Metabolomics enables characterization of small-molecule metabolites that reflect the physiological state of cells and tissues. As metabolites are downstream products of gene expression and protein activity, metabolomic profiling provides a direct assessment of cellular function and metabolic adaptation.

Heavy metal exposure is associated with disruptions in cellular energy production, amino acid and lipid homeostasis, oxidative stress biomarkers, and neurotransmitter-related pathways. These alterations may serve as early indicators of retinal dysfunction before the appearance of overt histopathological lesions.

Recent advances in systems biology have enabled the integration of transcriptomic, proteomic, and metabolomic datasets into multi-omics frameworks. These approaches support systems-level characterization of the biological responses underlying retinal toxicity, visual dysfunction, and regenerative processes. Integrating data across multiple levels of biological organization allows multi-omics analyses to identify molecular signatures associated with specific toxicants and to enhance mechanistic interpretation of retinal injury [60,61,63].

### 9.6. Future Perspectives for Biomarker Development

Identifying sensitive and reproducible biomarkers remains a key objective in environmental ocular toxicology, especially for the early detection of retinal injury before irreversible degeneration occurs. Integrating histopathological, immunohistochemical, behavioral, transcriptomic, proteomic, and metabolomic approaches offers significant potential for developing multidimensional biomarker panels that connect structural, molecular, and functional alterations resulting from heavy metal exposure. Collectively, these complementary methodologies provide a comprehensive framework for investigating retinal toxicity and regeneration in zebrafish, enhancing environmental risk assessment and supporting the development of preventive and therapeutic strategies for retinal disease.

## 10. Retinal Toxicity of Heavy Metals for Other Species and Translational Value for Humans

Evidence from non-zebrafish models indicates that several heavy metals affect conserved retinal structures and cellular pathways across vertebrate species. However, the magnitude and phenotype of toxicity vary according to species, developmental stage, exposure route, and experimental conditions. Cross-species comparisons should therefore be interpreted cautiously and used primarily to identify conserved mechanisms rather than to assume direct equivalence with human retinal disease.

Cadmium-related ocular toxicity has been demonstrated in several experimental systems. In lizard embryos, cadmium exposure caused impaired retinogenesis, including abnormal lens differentiation and optic cup development [64]. In rabbits, cadmium exposure produced ocular damage that included retinal and corneal alterations, with more pronounced effects reported during combined cadmium and lead exposure [65]. Electrophysiological studies in vertebrate photoreceptors have also shown dose-dependent impairment of rod-cell responses following cadmium exposure [66]. At the cellular level, studies using human retinal pigment epithelial cells have demonstrated that cadmium can activate endoplasmic reticulum stress and apoptotic signaling [67,68]. Human ocular tissue analyses have additionally reported altered elemental profiles in the choroid–RPE and retina in association with age-related macular degeneration, although such observational findings do not establish a direct causal relationship with cadmium exposure [69]. More broadly, cadmium-induced oxidative stress and disruption of antioxidant defense pathways have been described across experimental models and may contribute to retinal vulnerability [69,70,71].

Mercury-associated retinal toxicity has likewise been reported in both human and animal studies. In occupationally exposed workers, chronic mercury exposure has been associated with alterations in retinal nerve fiber layer, macular, and choroidal thickness [72]. Experimental studies in mice have demonstrated that prenatal mercury vapor exposure can result in mercury accumulation within the retina and optic nerve [73], while similar retinal deposition has been reported in squirrel monkeys following in utero exposure [74]. In the fish *Hoplias malabaricus*, dietary methylmercury exposure produced ultrastructural and morphological evidence of retinal neurotoxicity [75]. In rats, methylmercury exposure reduced electroretinogram (ERG) a- and b-wave amplitudes and increased retinal oxidative stress, whereas antioxidant-rich dietary intervention partially attenuated these effects [76]. Collectively, these studies support oxidative stress, mitochondrial dysfunction, and neuronal injury as recurrent mechanisms of mercury-associated retinal toxicity across species [73,75,76,77].

Lead-induced retinal injury has been studied predominantly in mammalian models. Experimental exposure in mice has been associated with photoreceptor and bipolar-cell loss, retinal apoptosis and necrosis, altered retinal vascular permeability, and long-term degenerative changes [8,78,79]. Reduced visually mediated responses have also been observed in lead-exposed animals, suggesting impairment of retinal and visual pathway function. Oxidative stress appears to contribute substantially to these effects, as lead exposure has been associated with reduced antioxidant enzyme activity and increased lipid peroxidation in rodent studies [80]. Together, these findings indicate that lead can affect both neuronal and vascular components of the retina.

Despite important interspecies differences, several common pathways emerge across zebrafish, mammalian, cellular, and human observational studies. Oxidative stress, apoptosis, inflammatory signaling, mitochondrial dysfunction, and retinal pigment epithelium injury recur across multiple exposure models. Zebrafish remain particularly valuable because of their conserved retinal organization, optical accessibility, rapid development, and capacity for functional visual assessment [81,82]. Nevertheless, their pronounced regenerative capacity and the absence of a true macula limit direct extrapolation to human retinal disease. Cross-species evidence should therefore be considered complementary, with zebrafish providing mechanistic and developmental insight that can be integrated with mammalian and human data to strengthen the translational interpretation of heavy metal-induced retinal toxicity.

## 11. Limitations, Knowledge Gaps, and Future Perspectives

Despite the growing body of evidence supporting the utility of zebrafish (*Danio rerio*) as a model for studying heavy metal-induced retinal toxicity, several limitations must be acknowledged when interpreting experimental results and extrapolating findings to human ocular disease. Zebrafish offer numerous experimental advantages, including rapid development, optical transparency, high fecundity, genetic tractability, and robust regenerative capacity. However, notable anatomical, physiological, and toxicokinetic differences exist between zebrafish and humans. Recognizing these limitations is crucial for accurately evaluating the translational relevance of zebrafish studies and for establishing priorities for future research.

### 11.1. Limitations of the Zebrafish Model

Although the zebrafish eye exhibits many structural and functional similarities to the human visual system, notable differences persist. Unlike humans, zebrafish lack a true macula and fovea, specialized retinal regions responsible for high-acuity central vision. Although cone-rich retinal areas with functional similarities to the human central retina have been identified, direct comparisons require cautious interpretation [10].

Differences in exposure routes constitute a significant limitation. In most zebrafish toxicology studies, heavy metals are administered via waterborne exposure, leading to continuous uptake through the gills, skin, and gastrointestinal tract. By contrast, human exposure typically occurs through dietary intake, inhalation, occupational contact, or chronic environmental exposure. These differences may influence toxicokinetics, tissue distribution, bioaccumulation patterns, and ultimately retinal toxicity.

Substantial heterogeneity among published studies presents an additional challenge. Experimental designs vary considerably in terms of exposure concentrations, developmental stage, exposure duration, analytical endpoints, and methodological approaches. This variability complicates direct comparison and may contribute to inconsistencies in reported findings. Furthermore, many studies employ concentrations that exceed environmentally relevant levels, which may limit both ecological and translational relevance.

The regenerative capacity of the zebrafish retina is both a major advantage and a potential limitation. Because retinal repair can occur rapidly following injury, the severity and persistence of retinal lesions observed in zebrafish may not accurately reflect outcomes in mammalian systems, where regenerative responses are substantially more limited [18,19,21]. Therefore, the dynamic balance between degeneration and regeneration should be carefully considered when interpreting toxicological findings.

Although histopathological assessment remains the most common endpoint in ocular toxicology studies, relatively few investigations simultaneously integrate structural, molecular, regenerative, and behavioral approaches. As a result, the relationship between retinal lesions, molecular alterations, visual dysfunction, and regenerative responses remains incompletely characterized for many heavy metals.

Important limitations are also related to:

- Model differences: zebrafish retinal cells regenerate readily while human retina has minimal regeneration capacity, and animal eyes lack a human-like macula, limiting extrapolation [82];
- Incomplete layer analysis: most studies focus on photoreceptor and ganglion cell layers, with the thin inner/outer nuclear and plexiform layers under-studied due to technical limits in tissue sectioning and immunolabeling;
- Neglected tissues: the cornea, lens, and optic nerve receive far less toxicological attention than the retina, despite their essential roles in vision;
- Proteomics lag: far fewer validated zebrafish-specific antibodies exist than for mammals, and low retinal protein yield limits quantitative proteomic study [60].

### 11.2. Major Knowledge Gaps

Although significant advancements have been made, critical knowledge gaps persist within the field of environmental ocular toxicology.

First, most available studies address embryonic and larval exposure, while the consequences of chronic adult exposure remain insufficiently characterized. Given that environmental contamination typically occurs over extended periods, understanding the cumulative effects of chronic low-dose exposure constitutes a major research priority.

Second, most investigations evaluate individual metals in isolation, even though aquatic organisms are generally exposed to complex contaminant mixtures comprising multiple metals, pesticides, pharmaceuticals, microplastics, and other environmental pollutants. Current evidence indicates that interactions among contaminants may produce additive, synergistic, or antagonistic effects. However, the implications of these interactions for retinal development, degeneration, and visual function remain poorly understood [35,36,37].

Third, relatively few studies have specifically examined retinal regeneration following heavy metal-induced injury. Determining how heavy metals influence Müller glia activation, progenitor cell formation, neuronal replacement, and retinal reconstruction represents an important area for future investigation [20,21,22].

Additional gaps include the limited understanding of emerging mechanisms such as ferroptosis, epigenetic regulation, mitochondrial dynamics, immune-mediated retinal injury, and metal-induced alterations of cell–cell communication within retinal tissues. Of these, ferroptosis has been identified as a major contributor to neurodegeneration through iron-dependent lipid peroxidation and oxidative membrane damage. Conversely, the involvement of the other pathways in heavy metal-induced retinal toxicity remains poorly characterized [42,53,81].

### 11.3. Emerging Technologies and Future Research Directions

Future research should prioritize environmentally realistic exposure paradigms that closely reflect conditions encountered in natural ecosystems and human populations. Chronic low-dose exposure models are particularly important, as they more accurately represent the cumulative effects of long-term environmental contamination compared to acute high-dose experiments.

Increased emphasis should also be placed on mixture toxicology. Investigating combinations of cadmium, lead, arsenic, chromium, copper, mercury, and co-occurring contaminants can provide a more realistic assessment of environmental risk and enhance understanding of complex toxicological interactions affecting retinal health.

Recent advances in molecular biology and systems toxicology offer significant opportunities for future research. Integrating transcriptomics, proteomics, metabolomics, epigenomics, and spatial molecular profiling with traditional histopathological assessment can facilitate the identification of novel biomarkers and improve mechanistic understanding of retinal injury. Multi-omics approaches may also distinguish adaptive responses from pathological processes and identify molecular signatures associated with susceptibility or resilience to toxicant exposure [60,61].

Artificial intelligence (AI) and automated image analysis constitute another promising area of development. Machine-learning algorithms can improve detection and quantification of subtle retinal lesions, facilitate high-throughput screening, and reduce observer-related variability in histopathological assessment.

Integrative multi-omics approaches provide opportunities to connect molecular alterations with histopathological and functional outcomes, enabling a systems-level understanding of toxicant-induced retinal pathology [60,61].

Future investigations should also focus on establishing stronger links between structural retinal injury and functional visual impairment. Combining histopathology, molecular analyses, regenerative biomarkers, and behavioral assays such as the optomotor response (OMR) and optokinetic reflex (OKR) can provide a more comprehensive assessment of retinal health and improve interpretation of toxicological findings.

Continued investigation of Müller glia-mediated retinal regeneration may have implications that extend beyond environmental toxicology. Elucidating the molecular mechanisms that enable zebrafish to regenerate retinal neurons following injury could contribute to the development of regenerative therapies for human retinal diseases characterized by irreversible neuronal loss, such as age-related macular degeneration, retinitis pigmentosa, diabetic retinopathy, and glaucoma [18,20,21].

Overall, zebrafish are expected to remain a central model in environmental ocular toxicology research. Addressing current limitations, adopting environmentally realistic exposure paradigms, integrating multi-omics technologies, and incorporating advanced computational approaches may substantially improve understanding of heavy metal-induced retinal injury, facilitate biomarker discovery, and accelerate the development of strategies aimed at preventing or treating environmentally associated visual disorders.

## 12. Final Considerations

Heavy metal contamination remains a major environmental challenge with considerable consequences for ocular health in aquatic organisms and humans. The reviewed evidence demonstrates that heavy metal exposure profoundly disrupts retinal development, structure, and function, leading to a wide range of pathological alterations, including developmental abnormalities, photoreceptor degeneration, retinal pigment epithelium dysfunction, visual impairment, and neurobehavioral disturbances. While individual metals exhibit distinct toxicological profiles, oxidative stress, mitochondrial dysfunction, apoptosis, neuroinflammation, and impaired retinogenesis consistently emerge as central, interconnected mechanisms underlying retinal injury.

Cadmium is the most extensively studied metal and consistently induces severe retinal toxicity, including retinal disorganization, vacuolization, photoreceptor degeneration, and developmental defects. Lead, arsenic, chromium, copper, mercury, and environmentally relevant contaminant mixtures also cause substantial retinal alterations through overlapping molecular pathways that converge on cellular stress responses and neuronal dysfunction. Notably, evidence indicates that functional visual deficits may precede observable structural degeneration, emphasizing the importance of integrating behavioral assessment with conventional histopathological and molecular analyses.

The zebrafish (*Danio rerio*) has emerged as a powerful and translationally relevant model for investigating heavy metal-induced retinal toxicity. Its conserved retinal architecture, cone-rich vision, rapid development, optical transparency, genetic tractability, and suitability for high-throughput experimentation enable comprehensive evaluation of retinal injury across developmental, histopathological, molecular, and functional levels. Additionally, the remarkable regenerative capacity of the zebrafish retina provides a unique opportunity to investigate endogenous mechanisms of neuronal repair and recovery, which are largely absent in mammalian systems. This distinctive feature allows for simultaneous investigation of both retinal degeneration and regeneration, thereby extending the utility of zebrafish beyond conventional toxicological assessment.

Advances in histopathology, immunohistochemistry, transcriptomics, proteomics, metabolomics, and integrated multi-omics approaches have substantially enhanced understanding of retinal responses to environmental contaminants. Emerging technologies, such as artificial intelligence-assisted pathology, automated image analysis, spatial molecular profiling, and systems toxicology, offer new opportunities to identify sensitive biomarkers of retinal injury and improve mechanistic interpretation of toxicological responses. Integrating these technologies with behavioral testing and regenerative biomarkers is expected to facilitate a more comprehensive understanding of the effects of environmental contaminants on retinal health.

Despite substantial progress, significant challenges persist. Current knowledge is largely derived from studies investigating acute exposure to individual contaminants, whereas real-world environmental conditions typically involve chronic low-dose exposure and complex contaminant mixtures. Future research should prioritize environmentally realistic exposure paradigms, longitudinal study designs, and multidimensional analytical approaches that can link molecular alterations with structural pathology, functional visual impairment, and regenerative outcomes.

Collectively, the available evidence establishes zebrafish as a highly valuable model for elucidating mechanisms of heavy metal-induced retinal injury, identifying biomarkers of toxicity, and exploring endogenous pathways of retinal repair. Continued investigation of the interactions among environmental contaminants, retinal degeneration, visual dysfunction, and regenerative responses will not only improve understanding of environmental ocular toxicology but may also provide important insights for the prevention, diagnosis, and treatment of human retinal diseases. Ultimately, zebrafish studies have the potential to contribute not only to environmental risk assessment but also to the development of future regenerative and precision-medicine strategies aimed at preserving vision and retinal function.

## Figures and Tables

**Table 1:** Key anatomical, physiological, and experimental characteristics that make zebrafish a suitable model for ocular toxicology research. Information compiled from [1,2,10,11,12,14,17,18,19,22,27].

Characteristic	Description	Relevance to Ocular Toxicology	References
Conserved retinal architecture	Retina organized into ONL, OPL, INL, IPL, GCL, and RPE, similar to other vertebrates	Enables translational investigation of retinal injury and degeneration	[10]
Cone-rich visual system	Zebrafish possess four cone photoreceptor subtypes (UV, S, M, and L cones) and tetrachromatic vision	Provides a visual system more comparable to human photopic vision than rod-dominated rodent models	[10,14]
Rapid ocular development	Eye formation begins within 24 h post-fertilization, with functional vision established by 4–5 days post-fertilization	Facilitates developmental toxicity studies and rapid screening of environmental contaminants	[10,17]
Optical transparency during development	Embryos and larvae are transparent, allowing direct visualization of retinal development in vivo	Enables non-invasive monitoring of ocular morphology and toxicant-induced abnormalities	[11,17]
High genetic homology with humans	Approximately 70% of human genes have at least one zebrafish ortholog	Supports investigation of conserved molecular pathways involved in retinal disease and toxicology	[10]
High fecundity and external fertilization	Large numbers of embryos can be obtained and exposed under controlled conditions	Suitable for high-throughput toxicological screening	[11]
Availability of transgenic lines	Numerous fluorescent reporter lines exist for retinal neurons, glia, and developmental pathways	Allows visualization of cellular responses to toxicants in vivo	[11]
Behavioral assessment tools	Established assays including optomotor response (OMR), optokinetic reflex (OKR), phototaxis, and startle response	Enables evaluation of functional visual impairment alongside structural pathology	[17,27]
Retinal regenerative capacity	Müller glia can dedifferentiate and generate retinal progenitor cells after injury	Unique model for studying retinal repair and regeneration following toxicant-induced damage	[18,19,22]
Environmental relevance	Aquatic habitat allows direct exposure to waterborne contaminants	Particularly suitable for ecotoxicological assessment of heavy metal pollution	[1,2,12]

Abbreviations: ONL, outer nuclear layer; OPL, outer plexiform layer; INL, inner nuclear layer; IPL, inner plexiform layer; GCL, ganglion cell layer; RPE, retinal pigment epithelium; UV, ultraviolet-sensitive; S, short-wavelength-sensitive; M, medium-wavelength-sensitive; L, long-wavelength-sensitive; OMR, optomotor response; OKR, optokinetic reflex.

**Table 2:** Histopathological, molecular, and functional alterations induced by heavy metals in the zebrafish retina.

Heavy Metal	Dosage Range and Reference	Major Histopathological Alterations	Principal Molecular Mechanisms	Functional Consequences
Cadmium (Cd)	100 µM/L [24,55] 0.3–3 mg/L [25] 17.8 µM [26] 0.5–2 ppb [36] 1 µg/L [37] 9 µM [36]	Microphthalmia, retinal disorganization, retinal thinning, photoreceptor degeneration, vacuolization, ganglion cell loss	Oxidative stress, mitochondrial dysfunction, ER stress, apoptosis, developmental gene dysregulation	Impaired visual behavior, altered locomotor activity, developmental visual deficits
Lead (Pb)	30 nM [23] 2.5–5 µg/L [34]	Retinal degeneration, retinal pigment epithelium damage, lens abnormalities, developmental retinal defects	Oxidative stress, calcium dysregulation, mitochondrial dysfunction, impaired neuronal differentiation	Reduced OMR performance, altered startle response, impaired visual processing
Arsenic (As)	300 ppb [28] 30–40 µM/L [51] 0.5–10 mM/L [54] 10–100 µg/L [53] 300 ppm [58]	Altered retinal architecture, RPE thinning, abnormal eye morphology, retinal developmental abnormalities	Oxidative stress, apoptosis, impaired neurogenesis, altered pax6a/ascl1a signaling	Behavioral abnormalities, impaired visually mediated responses
Hexavalent Chromium [Cr(VI)]	20–40 mg/L [29]	Retinal disorganization, nuclear pyknosis, retinal degeneration, lens-retina separation	ROS generation, DNA damage, apoptosis, oxidative stress	Reduced visual performance, developmental ocular defects
Copper (Cu)	0.8 µM/L [35] 3.9 µM/L [39]	Reduced retinal diameter, photoreceptor injury, ganglion cell loss, retinal thinning	Oxidative stress, ER stress, mitochondrial dysfunction, apoptosis	Visual impairment, developmental retinal abnormalities
Mercury (Hg)	30 ppb [1] 60 µg/L [56]	Retinal neurotoxicity, altered retinal organization, developmental abnormalities	Oxidative stress, mitochondrial dysfunction, calcium dysregulation, neuronal signaling disruption	Impaired visually mediated behaviors, neurodevelopmental deficits
Metal Mixtures	[35,36,54,55]	Enhanced retinal degeneration, developmental defects, increased oxidative injury	Combined oxidative stress, mitochondrial dysfunction, inflammatory signaling, altered developmental pathways	Greater visual dysfunction than individual metals; additive, synergistic, or antagonistic effects

Abbreviations: Cd, cadmium; Pb, lead; As, arsenic; Cr, chromium; Cr(VI), hexavalent chromium; Cu, copper; Hg, mercury; RPE, retinal pigment epithelium; ROS, reactive oxygen species; ER, endoplasmic reticulum; OMR, optomotor response; ppb, parts per billion; ppm, parts per million.

**Table 3:** Representative examples of heavy metal mixture toxicity in zebrafish, including exposure conditions, affected biological endpoints, proposed mechanisms, and reported interactions (synergistic, additive, or antagonistic).

Exposure Scenario	Developmental Stage	Major Affected Endpoints	Proposed Mechanisms	Reported Interaction	References	Evidence Strength
Cadmium (Cd) + Lead (Pb)	Embryos/larvae	Behavioral abnormalities, altered circadian activity, developmental toxicity, oxidative stress	Increased ROS generation, mitochondrial dysfunction, disruption of neurodevelopmental pathways	Additive to synergistic (endpoint-dependent)	[59,62]	**High**
Copper (Cu) + Cadmium (Cd)	Embryos/larvae	Growth impairment, developmental abnormalities, DNA damage, oxidative injury	Enhanced oxidative stress, impaired antioxidant defenses, mitochondrial dysfunction	Synergistic	[35]	**Moderate–High**
Cadmium (Cd) + Imidacloprid	Embryos/larvae	Retinal apoptosis, developmental toxicity, behavioral impairment	Nrf2/HO-1 dysregulation, oxidative stress, apoptotic activation	Synergistic	[36]	**Moderate**
Lead (Pb) + Cadmium (Cd) + Arsenic (As)	Early development	Neurodevelopmental alterations, visual impairment, delayed growth	Oxidative stress, DNA damage, developmental gene dysregulation	Additive	[1]	**Moderate**
Environmentally Relevant Heavy Metal Mixtures	Chronic exposure	Visual dysfunction, retinal degeneration, behavioral abnormalities	Chronic oxidative injury, neuroinflammation, cumulative toxicity	Context-dependent	[8]	**Limited–Moderate**
**Heavy Metals + Other Environmental Pollutants** (e.g., pesticides, pharmaceuticals, microplastics)	Embryos/larvae	Enhanced toxicity, developmental abnormalities, retinal dysfunction	Combined oxidative stress, inflammatory signaling, endocrine disruption	Frequently synergistic	[8,36]	**Moderate**

Abbreviations: Cd = cadmium; Pb = lead; Cu = copper; As = arsenic; ROS = reactive oxygen species; Nrf2 = nuclear factor erythroid 2-related factor 2; HO-1 = heme oxygenase-1.

**Table 4:** Representative histopathological, immunohistochemical, and molecular biomarkers used to investigate heavy metal-induced retinal toxicity in zebrafish. Information was compiled from representative experimental studies and reviews.

Category	Representative Biomarkers	Biological Significance	Applications in Retinal Toxicity Research	References
Histopathological Markers	Retinal thickness (ONL, INL, GCL), vacuolization, nuclear pyknosis, RPE integrity	Indicators of retinal structure and cellular damage	Assessment of retinal degeneration, tissue loss, and lesion severity	[24,25,29]
Oxidative Stress Markers	Nrf2, HO-1, SOD, CAT, 4-HNE, 8-OHdG	Cellular response to ROS generation and oxidative injury	Evaluation of oxidative stress, lipid peroxidation, and DNA damage induced by heavy metals	[1,7,8,28]
Apoptosis Markers	Caspase-3, Bax, Bcl-2, TUNEL	Regulation and detection of programmed cell death	Identification of retinal neuronal loss and toxicant-induced degeneration	[29,39,48]
Neuroinflammation Markers	GFAP, Iba-1, TNF-α, IL-1β, IL-6	Glial activation and inflammatory responses	Assessment of neuroinflammation associated with retinal injury	[7,8,35]
Proliferation and Regeneration Markers	PCNA, Ki67, BrdU, Ascl1a, Lin28	Cellular proliferation and Müller glia-mediated regeneration	Evaluation of retinal repair and regenerative responses following injury	[18,20,21,22]
Developmental and Transcriptomic Markers	Pax6, Sox2, Opsins, pax6a, ascl1a, nrf2, bax	Retinal development, photoreceptor differentiation, and molecular responses to injury	Investigation of developmental toxicity, retinal maturation, and mechanistic pathways	[1,13,28,29]

Abbreviations: ONL = outer nuclear layer; INL = inner nuclear layer; GCL = ganglion cell layer; RPE = retinal pigment epithelium; Nrf2 = nuclear factor erythroid 2-related factor 2; HO-1 = heme oxygenase-1; SOD = superoxide dismutase; CAT = catalase; 4-HNE = 4-hydroxynonenal; 8-OHdG = 8-hydroxy-2′-deoxyguanosine; ROS = reactive oxygen species; TUNEL = terminal deoxynucleotidyl transferase dUTP nick-end labeling; GFAP = glial fibrillary acidic protein; Iba-1 = ionized calcium-binding adaptor molecule 1; TNF-α = tumor necrosis factor-α; IL-1β = interleukin-1β; IL-6 = interleukin-6; PCNA = proliferating cell nuclear antigen; BrdU = bromodeoxyuridine.

## Data Availability

No new data were created or analyzed in this study. Data sharing is not applicable to this article.
